# Striatal response to negative feedback in a stop signal task operates as a multi-value learning signal

**DOI:** 10.1162/imag_a_00024

**Published:** 2023-11-03

**Authors:** Benjamin J. Smith, Megan Lipsett, Danielle Cosme, Victoria A. Braun, Anastasia M. Browning O’Hagan, Elliot T. Berkman

**Affiliations:** Center for Translational Neuroscience, University of Oregon, Eugene, OR, United States; Annenberg School for Communication, University of Pennsylvania, Philadelphia, PA, United States

**Keywords:** striatum, reinforcement learning, multi-value, stop signal task

## Abstract

*Background and aim:* We examined error-driven learning in fMRI activity of 217 subjects in a stop signal task to obtain a more robust characterization of the relation between behavioral measures of learning and corresponding neural learning signals than previously possible.

*Methods:* The stop signal task is a two-alternative forced choice in which participants respond to an arrow by pressing a left or right button but must inhibit that response on 1 in 7 trials when cued by an auditory “stop signal.” We examined post-error learning by comparing brain activity (BOLD signal) and behavioral responses on trials preceded by successful (correct stop) vs. failed (failed stop) inhibition.

*Results:* There was strong evidence of greater bilateral striatal activity in the period immediately following correct (vs. failed) stop trials (most evident in the putamen; peak MNI coordinates [-26 8 -2], 430 voxels, p < 0.001; [24 14 0], 527 voxels, p < 0.001). We measured median activity in the bilateral striatal cluster following every failed stop and correct stop trial and correlated it with learning signals for (a) probability and (b) latency of the stop signal. In a mixed-effects model predicting activity 5-10 s after the stop signal, both reaction time (RT) change (B = -0.05, t = 3.0, χ^2^ = 11.3, p < 0.001) and probability of stop trial change (B = 1.53, t = 6.0, χ^2^ = 43.0, p < 0.001) had significant within-subjects effects on median activity. In a similar mixed model predicting activity 1-5 s after the stop signal, only probability of stop trial change was predictive.

*Conclusions:* A mixed-effects model indicates the striatal activity might be a learning signal that encodes reaction time change and the current expected probability of a stop trial occuring. This extends existing evidence that the striatum encodes a reward prediction error signal for learning within the stop signal task, and demonstrates for the first time that this signal seems to encode both change in stop signal probability and in stop signal delay.

## 1 Introduction

Reinforcement learning (RL) is a mechanism for an agent to maximize expected reward by calibrating behavior to match behaviors that have been reinforced with reward (or punishment) in the past (Sutton et al., 1992). RL has directly measurable signals in neural circuitry (Schultz et al., 1997), has been foundational for the development of our understanding of human learning in general (Shteingart & Loewenstein, 2014), and not only underpins human learning but also seems fundamental for the development of human-level artificial general intelligence (Ide et al., 2022; Silver et al., 2021; Vamplew et al., 2022). RL is also important in the development of appropriate response inhibition, which plays a key role in goal-directed behavior (Berkman, 2018; Verbruggen & Logan, 2008), psychopathological conditions (Howlett et al., 2023), and in inhibitory response training for reducing unhealthy food intake (Houben, 2011; Lawrence et al., 2015). How can a habitual inhibitory response be deliberately induced? There is evidence that inhibitory responses can in fact be trained (Verbruggen & Logan, 2008); RL models are important for explaining how this training occurs. In fact, most inhibitory responses originate from a behavioral association, even if they are cognitively driven. How do those inhibitory responses arise?

The Stop Signal Task (SST) (Aron & Poldrack, 2006) is a widely used reaction-inhibition task used to assess an individual's ability to control their response in the face of an external stimulus. Subjects are instructed to respond as quickly as possible to the stimulus in each trial, unless a tone, the “stop signal,” is played, in which case subjects have been instructed to inhibit responding. The timing of the tone, the "stop signal delay" (SSD) is titrated between trials based on individual performance to create a challenge for the subject—shorter SSDs make it harder to correctly inhibit a response to the arrow—so that most subjects achieve around 50% correct performance on stop trials.

Response inhibition in SST is a type of RL that can be learned over the course of the task (Verbruggen & Logan, 2008). Subjects must learn to expect a tone in a particular amount of time, then relearn when the tones change as the algorithm adjusts the stop signal delay in order to maintain a difficulty level that yields 50-50 performance rate. During SST, there are at least two kinds of learning: stop trial probability and stop signal delay on stop trials. Every trial is potentially an update on the likelihood of stop trials occurring, that is, stop trial probability, or P(Stop). Subjects must learn the stop signal delay (SSD) given the current trial is a stop trial. P(Stop) and SSD are both predictable features that the SST subjects can learn to improve their performance. Accordingly, a natural hypothesis is that reward prediction error (RPE)-related dopaminergic brain activity would be evident during the SST task, and that it would track changes in expected P(Stop) and SSD.

The SST task can be informative about how both errors and correct responses influence subsequent behavior and its neural correlates. In particular, studying learning processes in the SST task can advance our understanding of learning processes related to response inhibition. “Post-error slowing” in the SST refers to increases in response time on trials following an error (compared to a correct response) and reflects an adaptive learning process by which participants adjust behavior within environments of low predictability (Bissett & Logan, 2012).

Several authors have previously explored computational models of learning in the stop signal task, and we mention just a few here. Chevrier and Schachar (2010) described error detection in the SST, within a reinforcement-learning pathway, and found post-error slowing deactivations in the striatum in a sample of 14 healthy subjects. The striatum modulates dopamine output and encodes error magnitude, which led Chevrier and Schachar to suggest the striatal deactivation in fact functions as a learning signal in post-error slowing. However, this previous work was limited in power (N = 14) and did not examine two distinct forms of learning within the SST, but focused solely on the stop signal delay learning. Ide et al. (2013) found, using a Bayesian ideal observer model, that the dorsal anterior cingulate cortex tracks absolute prediction error of stimulus expectation vs. outcome, and signed prediction error related to response outcome, and Hu et al. (2015) found mPFC activity tracks stop signal likelihood.

Understanding how two simultaneous forms of learning—P(Stop) and SSD—occur within the same task represents a step forward in modeling learning in the SST. The present study is by far the most powerful and comprehensive examination of reward learning in the SST. Furthermore, the present study is the first to demonstrate how a computational model of learning can model the learning of multiple features—P(stop) and SSD—simultaneously.

Training inhibition via RL is important for applied behavioral psychology where the frequency of undesired behaviors must be reduced, such as in reducing unhealthy food consumption. In food consumption inhibition training, reduced energy intake has been induced by presenting conditioned response food images paired with “no go” signals training participants to withhold a response (Houben, 2011; Lawrence et al., 2015). However, RL processes during these tasks have not been explored in the context of probabilistic inhibition such as that occurring in the SST task. Better understanding the neural-computational mechanisms of inhibitory training can better help us understand learning in applied habit contexts.

### 1.1 Reward prediction error

We examined whether activity in the brain represented reward prediction error as a test of the computations implied by our model. We focused on activity in the striatum because phasic dopaminergic activity in the ventral tegmental area and substantia nigra encodes reward prediction error (Schultz, 2022; Schultz et al., 1997) and projects primarily to the NAcc (Lerner et al., 2021). In a neurosynth (Yarkoni et al., 2011) automated meta-analysis, “reward” is generally related to an intersection of the accumbens and putamen within the striatum. We also considered activity in the frontal cortical cortex and the anterior cingulate as comparator regions. The anterior cingulate cortex is involved in conflict monitoring, and as such, we expect it to respond to negative feedback, while we expect the frontal orbital cortex to respond to value signals, but we expect neither to carry RL signals in both positive and negative directions.

### 1.2 Hypotheses

The role of the striatum in reward prediction error, as described above, is well established. What exactly must subjects predict in order to perform optimally in the SST task? Above, we identified two parameters that must be predicted: SSD and stop signal cue probability. It is well established that reward prediction errors in the stop signal task tracks stop signal cue probability (Chevrier & Schachar, 2010; Ide et al., 2013), but to our knowledge this is the first time it has been proposed that subjects also track the changing Stop Signal Delay as it is adjusted to achieve a specific level of performance in the task. As argued above, anticipating the length of the Stop Signal Delay helps participants understand how long to wait before initiating a Go response due to accumulating evidence that the trial is a Go trial.

Accordingly, we hypothesize that the striatum carries an RL neural signal following the stop signal cue in the SST that calibrates expectations of the (1) stop signal delay and (2) stop signal cue probability. Based on this hypothesis, we predict that

1. Following Correct vs. Failed Stop trials, we will see more striatal activity.
2. Correct Go and Correct Stop activity will move in opposite directions, indicating a reward prediction error, where Correct Go represents better than expected activity and Correct Stop represents worse than expected activity. This activity will occur in regions associated with reward—principally the striatum—but not other regions in the prefrontal cortex including the frontal orbital cortex (FOC) and anterior cingulate cortex (ACC) which are associated with other aspects of the task.
3. Because the striatal signal in the post-Correct vs. Failed Stop activity represents an RPE, it should correlate with change in expected stop signal delay, change in expected stop signal likelihood, or both.

## 2 Materials and Methods

### 2.1 Participants

We collected data from participants as part of a wider intervention study on healthy eating; primary inclusion criteria were aged between 18 and 60, a BMI over 25, and having an interest in eating more healthfully. Human subjects research was approved by the University of Oregon IRB under protocol number 04282017.047. SST task data were collected in two sessions for each subject. We used data from the baseline session before participants were randomized to intervention conditions to extract an ROI and measure activity in that ROI across data in both sessions. Of 275 subjects with SST sessions, 20 were removed due to irregularities in the task procedure noted at the time of scan. An additional four were removed due to missing scan data, and an additional 34 were removed due to fMRI data quality issues—primarily motion or ghosting artifacts—leaving 217 participants’ data to be analyzed.

Of those 217 participants, ages ranged 18-60 years old M(SD) = 37(11), including 24% Male Assigned At Birth (AAB), 75% Female AAB, and the remainder not recorded. Racial and ethnic identifications were as follows: White 82%, Hispanic or Latino 9%, More Than One Race 5%, Asian 3%, American Indian/Alaska Native 2%, Black or African American 2%, and Race Unknown or Not Responded 2%.

### 2.2 Neuroimaging data acquisition and preprocessing

Neuroimaging data were acquired on either a 3T Siemens Allegra or Skyra scanner at the University of Oregon Lewis Center for Neuroimaging. Results included in this manuscript come from preprocessing performed using fMRIPrep 22.1.1 (Esteban et al., 2018). Scan sequence parameters and preprocessing details appear in Supplementary Material, but briefly, anatomical images were segmented and normalized to MNI space using FreeSurfer (Fischl, 2012); functional images were susceptibility distortion corrected, realigned, and coregistered to the normalized anatomical images. Normalized functional data were then smoothed (6 mm^3^ FWHM) in SPM12. Motion estimation from fMRIPrep was used to correct for motion artifacts. A machine-learning classifier trained on prior data was used to identify motion artifacts and exclude these images (Cosme et al., 2018); more information is available in the Supplementary Material.

### 2.3 Task description

**Fig. 1.**
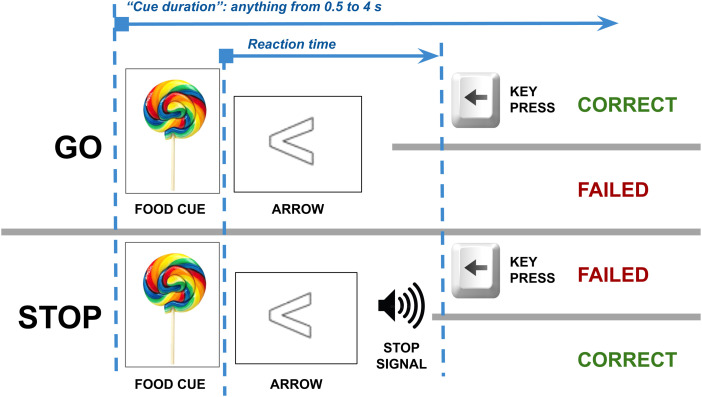
Stop Signal task. Each trial is a Go trial or a Stop trial; approximately 1 in 7 trials are a Stop trial; so, in 6 of 7 trials, a participant can safely press a key corresponding to the arrow shown on screen to get the trial correct. Participants must watch for the 1 in 7 trials where they must inhibit the key press after hearing the stop signal. Timing is calibrated so that the participant gets 50% of Stop trials wrong.

In our SST (Aron & Poldrack, 2006; Logan, 1994), subjects respond to an arrow by pressing a left or right button, but in 15% of trials an auditory “stop signal” was played shortly after the appearance of the arrow, cueing participants to inhibit the response (Fig. 1). The task has 128 trials, with difficulty adjusted via automatic timing of the tone to ensure a mix of correct and incorrect responses. It also contains food cues, to test healthy eating behavior hypotheses that are not addressed in this paper. The task is available for download at https://github.com/UOSAN/SST_DEV.

### 2.4 Measures

ΔSSD is operationalized as the difference between the reaction time on the current trial and the reaction time on the following trial, that is, the empirical change in the subject’s reaction time coinciding with the learning signal trial. To model P(Stop), for each trial, we calculated the proportion of all prior trials in the task that have been stop trials. This proportion will fluctuate a lot during initial portions of the task, and then settle into a fairly narrow band late in the task because each successive trial has a smaller effect on the total proportion of stop trials as the total number of trials increases. We chose this method because it best represents what an “unboundedly rational” subject would use to estimate the proportion of stop trials in the task, given the variable ratio schedule of trials that contain a stop signal. RPE (as in Equation 1) will be negative during a Failed Stop trial, positive during a Correct Stop trial, and relatively neutral during a Correct Go trial. The SST is designed so that around 50% of all Stop trials are correct and 50% are incorrect, and so, in Failed and Correct Stop trials, we can expect relatively equal but opposite reward prediction error signals.

### 2.5 Modeling the task

In RL, RPE is the difference between the expected reward and the actual reward received after an action is taken (i.e., the feedback) (Schultz, 2022; Schultz et al., 1997). The RPE is used to update the agent's value function, which represents its knowledge about the expected reward for each state-action pair. We can model an RPE signal in the SST. Because subjects might attempt to learn both probability of stop trial and stop signal delay time, we hypothesize that there are brain regions that signal an RPE in response to updates to either of those signals. This suggests a simple linear model predicting RPE in the SST:

$$RPE=\beta_{1}\Delta \text{P}\left(\text{Stop}\right)+\beta_{2}\Delta \text{SSD}+\varepsilon$$ (1)

where P(Stop) is the expected probability of a trial being a stop trial, SSD is the expected duration of a stop signal delay given that a trial is a stop trial, and Δ represents the change in each of these in response to feedback during a trial.

## 3 Task Contrast: Neural Activity in the Striatum Distinguishes Correct Stop from Failed Stop Trials

We wanted to test H1, that reward activity in the striatum would be evident following Correct Stop (vs. Failed Stop) trials. More broadly, we wanted to understand the mechanisms through which participants observed Stop signals, how they responded to information about them (immediately following the signal), and what they did with that information (during the next trial). Post-error slowing in the trial following a Stop error is a widely observed phenomenon in Stop Signal Tasks, and we wanted to more carefully understand the neural mechanisms of post-error slowing. Consequently, we contrasted Correct vs. Failed Stop trials at three periods: (a) during the trial itself; (b) during the inter-trial interval (ITI) following the trial, and (c) during the following trial.

### 3.1 Methods

Event-related condition effects were estimated in first-level analyses using a fixed-effects general linear model and a canonical hemodynamic response function. Regressors modeled each experimental condition. More information is available in the Supplementary Material, but in brief, three separate SPM models described respectively bidirectional contrasts of Correct Stop vs. Failed trials, Go trials following those two trial types, and ITIs following those two trial types.

#### 3.1.1 Second-level contrasts

All second-level analyses then used an SPM one-sample t-test to find significant activity patterns across all subjects that contained each contrast. There were 217 subjects in each contrast. Based on the second-level analysis described above, we extracted contrast maps describing activity clusters at the three time points of interest. Contrasts were extracted only from the first of two waves of data collection. In detecting clusters, reported p-values were subject to family-wise error correction.

### 3.2 Results

**Fig. 2.**
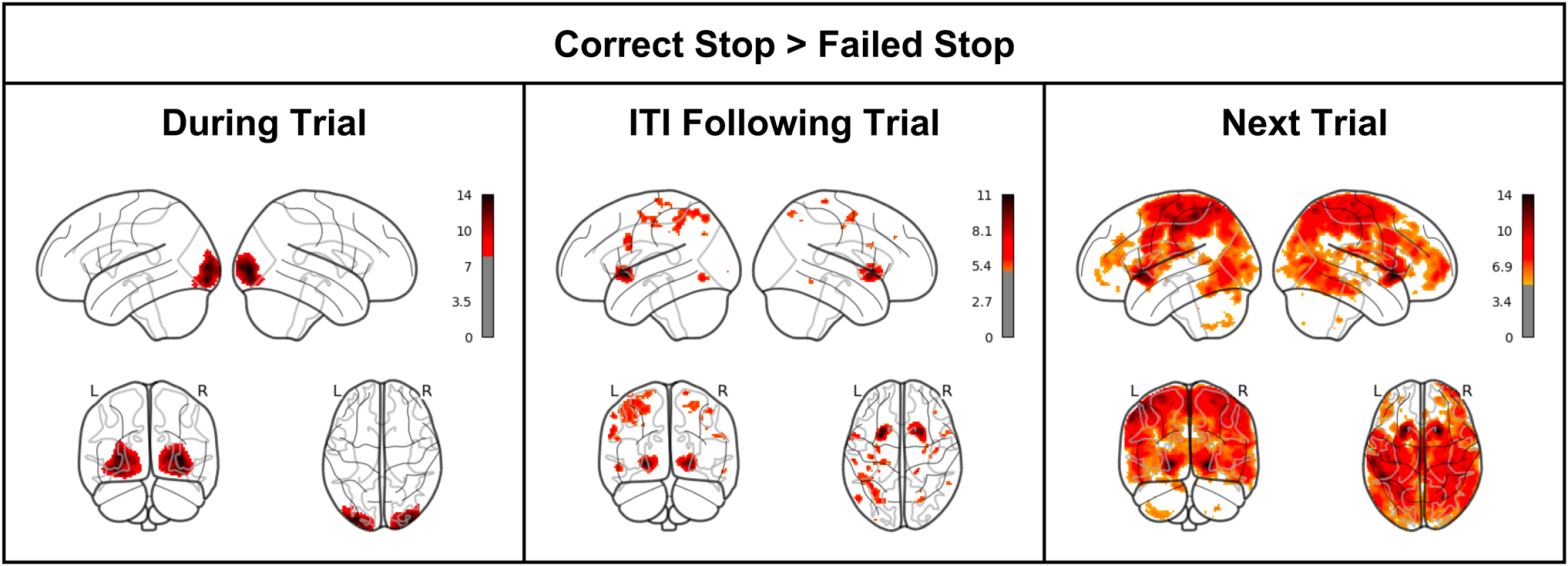
Statistical parametric map contrast of Correct Stop vs. Failed Stop (1) during stop trials (i.e., the activity pictured in Fig. 1) (2) during the interval following the trial (i.e., the time after the stop trial and before the next trial) and (3) in the next trial (as participants make a decision having updated on information in the current trial).

In the ITI Following Correct Stop > Failed Stop contrast (Fig. 2), there was strong evidence of greater bilateral striatal activity (most evident in the putamen; peak MNI coordinates [-26 8 -2], 430 voxels, FWE-corrected p < 0.001; [24 14 0], 527 voxels, FWE-corrected p < 0.001). Significant clusters of striatal activity were also evident in a whole-brain contrast in the following trial. There were no significant clusters of activity in the reverse ITI Following Failed Stop > Correct Stop contrast.

### 3.3 Discussion

In a Correct Stop > Failed Stop contrast—both in the ITI phase following the trial, and in the subsequent trial—there exists a strong striatal signal, consistent with the first hypothesis of greater striatal signal after Correct Stop trials. This signal does not appear to be present during the trial itself, but instead, appears following the trial. It is unclear whether this is due to a decrease in striatal activity following Failed Stop trials, or an increase in striatal activity following Correct Stop trials.

There is evidence that deactivation occurs in post-error slowing (Chevrier & Schachar, 2010), but the striatum is typically associated with reward prediction error in the positive domain, including unexpected reward, or even simply reward, unpredicted or not (Niv, 2009). In the SST, striatal activity has been linked to stop-signal probability (Zandbelt & Vink, 2010).

Activity contrast in the next trial is most widespread relative to the primary trial and ITI. It is most concentrated in the striatum and motor cortex. It is plausible that one consequence of the widely observed post-error slowing phenomenon is less motor cortex activity in the trial as responding slows, but this is speculative.

## 4 Failed and Correct Stop Neural Responses to the Tone

Consequently, we examined activity within each condition to determine if the observed striatal activity contrast is better characterized as an increase in activity following Correct Stop trials, or a decrease in activity following Failed Stop trials, or both. This analysis enabled us to resolve two specific questions. First: is the observed striatal contrast due to Correct Stop activation or Failed Stop deactivation? These were not discernable in trial type beta images separately recorded for Correct Stop and Failed Stop trials because striatal activity was apparent in both trial type beta images. Second, another pattern observed in the task contrast (Fig. 1) was that activity seemed to “evolve” from during the trial of interest, to the cue, and then into the next trial, with primarily occipital cortex activity pre-tone, striatal activity following the trial, and more broadly distributed activity including the striatum in trials following Correct vs. Failed Stop trials. How was this related to the activity over time?

To investigate these questions, we next extracted BOLD activity in specific anatomical ROIs over time, relative to the stop signal, to examine Correct Stop and Failed Stop separately, while better understanding their evolution over time and the part of the striatum from which they derived. We extracted purely anatomical ROIs in order to understand whether three components of the striatum (accumbens, putamen, and caudate) dissociated, and whether the FOC (Hu et al., 2015) and ACC (Ide et al., 2013), which are also involved in SST learning, dissociate from striatal components. The RPE hypothesis is more consistent with highly correlated striatal ROIs across the Correct Stop and Correct Go trials, contrasted with less correlated activity in comparator areas such as the ACC and frontal orbital cortex. Conversely, differential roles for those striatal components have been previously identified in the SST specifically (Chevrier & Schachar, 2010) and in learning generally (Brovelli et al., 2011; Mizumori et al., 1999), andi if striatal components dissociate from one another, this may be suggestive of specialist roles in, for example, action initiation (Suzuki et al., 2021).

### 4.1 Methods

To understand where the activity might be coming from, and to localize the effects, we examined activity over time from the moment of the tone and into the next trial. This enabled us to see where the trials peaked.

First, at each time point, we extracted the average of activity across all voxels in each of the following four ROIs: (a) subregions of the striatum, that is, the caudate, putamen, and accumbens, using FSL’s (McCarthy, 2023) Harvard-Oxford Subcortical atlas (Kennedy et al., 2016), and (b) the functional ROI determined by the area in the “ITI Following Trial” contrast in the previous analysis. Time series representing ROI activities are then mean centered and normalized by subtracting the mean across all time series at each time point from all time series.

Then, we estimated the BOLD response by sampling activity in 0.1 s bins in the range from 10 s prior to the stop signal delay to 20 s following it. Each bin included every 2 s TR image that overlapped with the bin. This yielded a fine-grained description of BOLD response similar to EEG or a more precise Finite Impulse Response. For Go trials, which do not have stop signal delays, we estimated an expected stop signal delay based on the last stop trial and extracted a similar 30 s window around that period.

We applied this FIR-like method because there are specific sets of responses we wanted to capture—signal salience, signal reaction, learning reaction, next trial behavior—and the latency of these events relative to the stop signal is not exactly clear. By using an FIR-like time course, we can identify relevant responses without pre-specifying an arbitrary predetermined time.

Data were collected from two waves of the SST task. Because the task is calibrated so that participants should get approximately 50% of all stop trials correct, extremely high or low performance is indicative of some problem with the task, or of participant disengagement. We therefore excluded 23 runs where the proportion correct was less than 20%, and 2 where the proportion correct was higher than 80%. Overall, 5 subjects were excluded, leaving 217 subjects for the analysis. See Figure in the Supplementary Material for more details.

### 4.2 Results

**Fig. 3.**
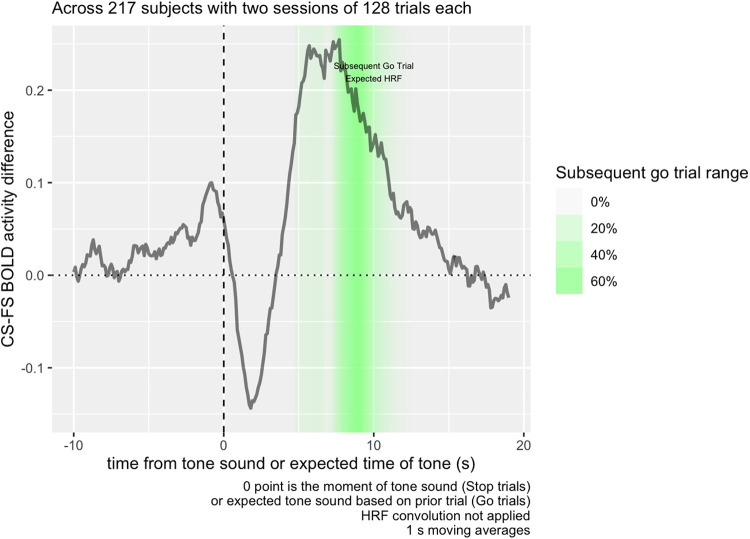
Raw mean BOLD activity contrast in the inter-trial ROI from Figure 1, following Correct vs. Failed Stop trials, averaged across 217 subjects with two sessions of 128 trials each. Following the stop signal, BOLD contrast decreases, but quickly increases at the 5-10 s mark. Activity extends into the presentation of the subsequent Go Trial; accounting for a standard HRF delay of 4-6 s, we would expect to see BOLD activity related to that subsequent trial in the highlighted region in the figure.

Peak BOLD activity, as modeled in SPM’s HRF, occurs 4-6 s after an event itself, and where Go trials follow a stop signal, they tend to occur about 4 s following a stop signal in the previous trial, so BOLD activity used in a contrast of the Go trial following a stop signal should occur around 8-10 s, the time point highlighted in Figure 3, and BOLD activity in the ITI period should occur immediately before that.

Considering where the canonical HRF function predicts activity corresponding to the ITI and subsequent period, we can see from Figure 3 how the Correct Stop > Failed Stop contrast in the striatal region is strongly positive at the 5-10 s point, explaining the striatal contrast at this time in Figure 1. We also see that there is a sharp decrease in contrast in the same area immediately after the Stop Signal. How much of each of these components is due to Correct Stop or Failed Stop activity?

**Fig. 4.**
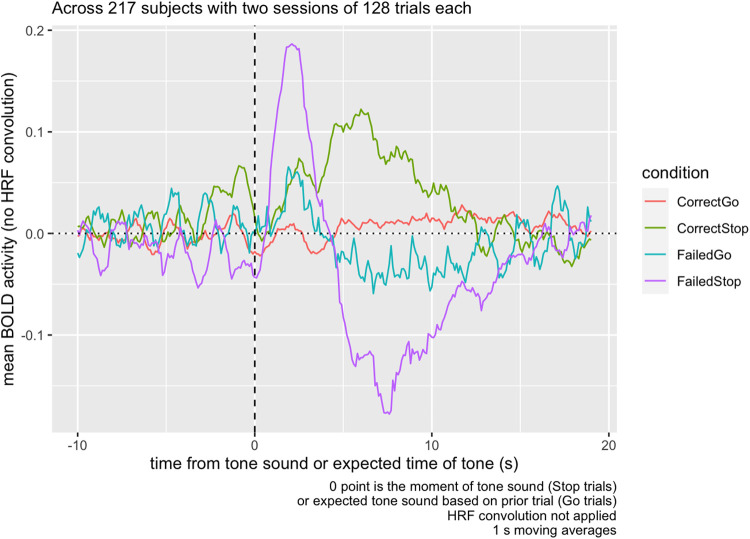
Raw mean BOLD activity in the inter-trial ROI from Figure 1, by trial type, averaged across 217 subjects with two sessions of 128 trials each. The ROI responds to the tone, but in opposite directions depending on whether the participant correctly responded to it or not. In Go trials, there is no discernible response.

Breaking up this activity separately into Correct Stop and Failed Stop trials (Fig. 4), we can see that the activity observed in the contrasts in the Task Contrast decomposes into a decrease in functional activity during the Failed Stop trial, and an increase in the Correct Stop trial. The sharp contrast decrease immediately after the tone depicted in Figure 3 is in fact an *increase* in activity immediately following the Stop Signal in the Failed Stop condition. This sharp decrease is not apparent in Correct Stop trials.

**Fig. 5.**
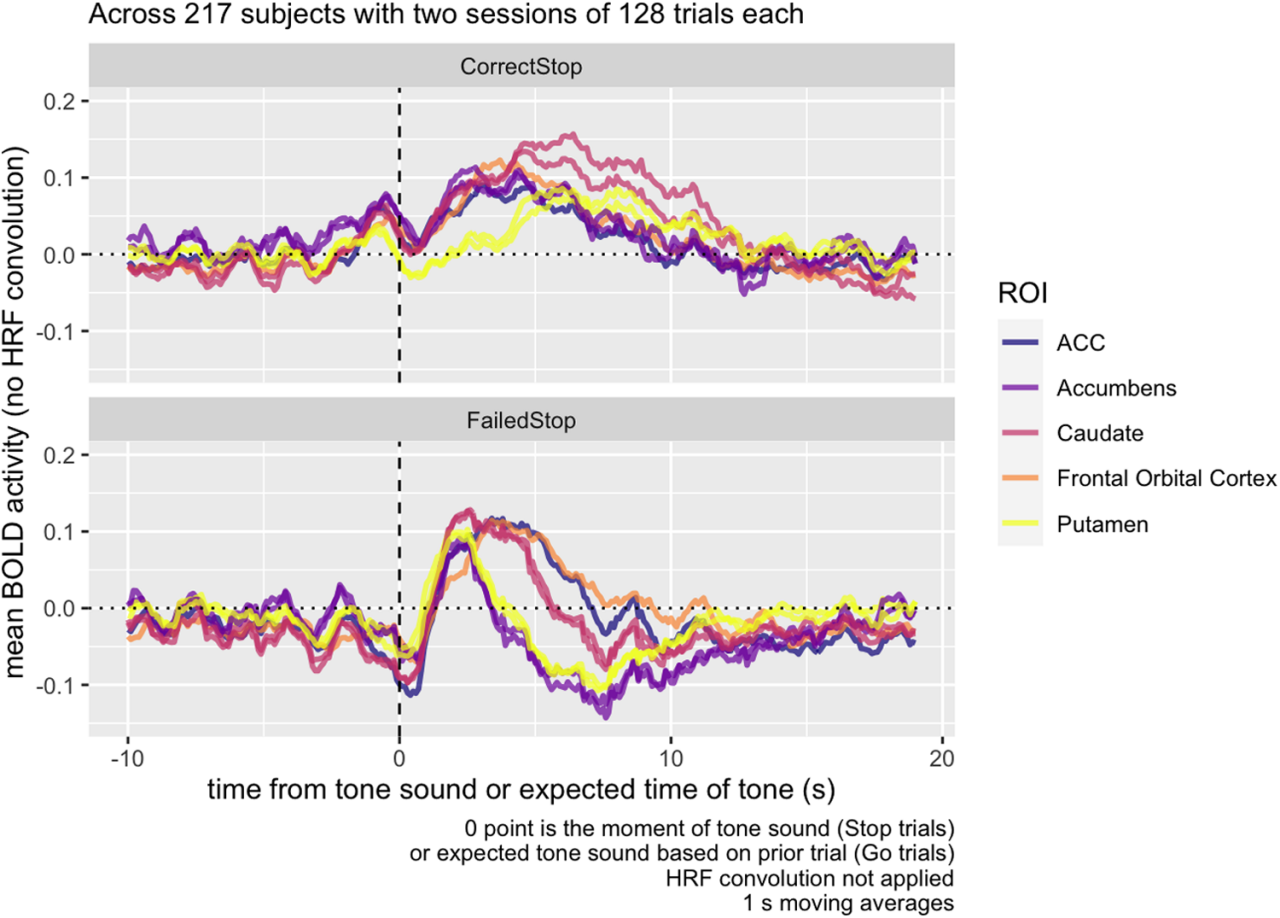
Raw mean BOLD activity in the inter-trial ROI from Figure 1, by trial type, averaged across 217 subjects with two sessions of 128 trials each. At the 5-10 s post-stop-signal point, accumbens and caudate show elevated activity during Correct Stop trials, while putamen and accumbens show depressed activity at the same time period in Failed Stop trials.

Examining the anatomical ROIs, across all regions measured, baseline activity was relatively constant and typically in the range of -0.1 to 0.05. In Failed Stop trials, all regions show a peak of activity at around 4-5 s following the Stop signal (Fig. 5). Putamen and accumbens then show a *decrease* in activity around the 8 s mark. In contrast, in Correct Stop trials, there is an increase in all measured regions accumbens, caudate, ACC, and FOC activity in the 5 s following the stop signal, but putamen activity does not exceed baseline until more than 5 s following the Stop Signal, and reaches a much lower peak than other areas.

**Fig. 6.**
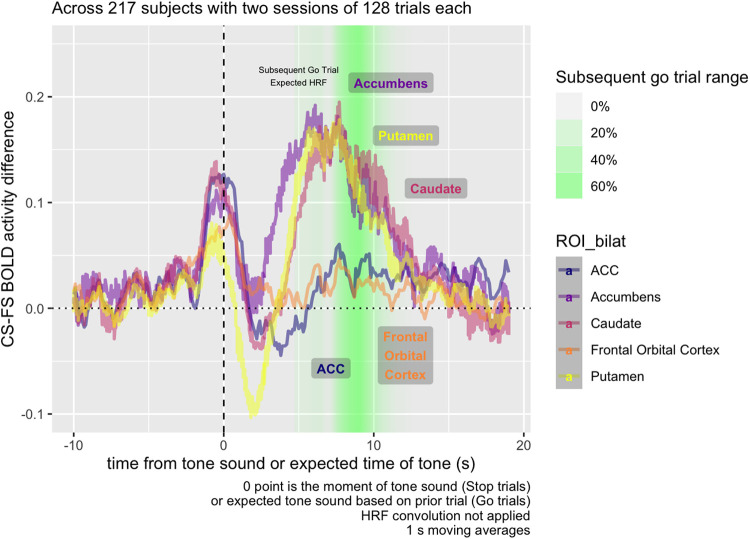
Raw BOLD mean activity contrast in five different bilateral ROIs, including three within the striatum (accumbens, caudate, and putamen) and two cortical ROIs (ACC, frontal orbital cortex). In striatal ROIs, but not cortical ROIs, BOLD activity contrast is higher 5-10 s following the Stop signal on Correct Stop compared to Failed Stop trials.

Figure 6 examines the Correct vs. Failed Stop difference, breaking down activity by region. While the dip in activity at t = 0, during Failed Stop, occurs for all regions, relative to prior to the Stop Signal, the subsequent RPE at the 5-10 s mark only appears for striatal areas—the accumbens, caudate, and putamen—and not the ACC or FOC.

### 4.3 Discussion

We found evidence of two distinct post-stop signals: a salience signal immediately following a Failed Stop signal, and a subsequent valenced Failed vs. Correct Stop signal carrying RPE information. The first salience signal is a spike in BOLD response (i.e., not adjusted for the HRF) across all regions measured, 1-3 s following the Failed Stop signal (Fig. 5). The second signal is a non-HRF-adjusted-BOLD response largely restricted to the striatal regions, in the 4-10 s following the Stop Signal (Fig. 6), positive in Correct Stop trials and negative in Failed Stop trials.

The second signal in the 4-10 s window resembles an RPE signal. The time courses for the Failed Stop trial suggest a strong negative reaction to a missed Stop signal in the Putamen and Accumbens, but not in the Caudate, FOC, or ACC. Conversely, time courses in the Correct Stop condition suggest all three striatal subregions and both of the other two regions examined (FOC and ACC) respond positively. Furthermore, the Caudate’s Correct Stop signal is particularly strong. The net effect visible in Figure 6 is that all three striatal regions indicate a differential RPE signal for Correct vs. Failed Stop, although this comprised different signals in each area. For the Accumens and Putamen, the signal is truly valenced: negative following a Failed Stop and positive following a Correct Stop. For the caudate, there is still a differential RPE signal, but this consists of a strong positive response to Correct Stop and a null response to Failed Stop.

The first signal in the 1-3 s window is suggestive of a salience signal. Typically, a peak BOLD response is expected 4-6 s after associated neural activity, so the peak of 1-3 s observed in the Failed Stop condition across all five regions might suggest neural activity occurring *prior* to the stop signal. This would be difficult to explain, because 1-3 s spike occurs only in Failed Stop trials, so participants would have to be anticipating a Stop failure. Chen et al. (2021) have demonstrated that lower-intensity stimulation elicits narrower and faster HRFs than normally expected, and perhaps this particular response simply occurs more quickly than the typical 4-6 s.

#### 4.3.1 Reward prediction signal

The contrast observed in Task Contrast for the Subsequent Go Trial is mainly derived from the period 8-10 s following the Stop Signal. Figure 3 shows that this time period decomposes into both a decrease in striatal activity during Failed Stop trials, and an increase in Correct Stop trials. Considering that the error rate for both Failed Stop and Correct Stop trials is algorithmically adjusted towards 50%, in a standard RL model, these should be about equally informative. Consequently, both should have about equal and opposite RPE signals. This is in fact what we are seeing in the period 5-10 s from the Stop Signal and is therefore consistent with these signals indexing RPE.

## 5 Dual Learning Signal Analysis: Striatal Neural Activity Encodes Response Change

Next, we sought to test whether the neural signals identified above fit behavioral learning during the task. This could help us confirm their role in learning as described in our multi-feature computational model in Equation 1, predicting RPE as a combination of Change in Expected P(Stop) (ΔP(Stop)) and Change in Reaction Time (ΔRT) as an operationalization for Change in Expected SSD (equivalent to Post-Stop Slowing, although slowing does not occur in absolutely every trial). Optimal performance on the task would be achieved if subjects behave based on a correctly estimated expected P(Stop) and expected SSD. For each trial, we extracted a set of response magnitude estimates, including peak, trough, and median activity, from the functionally defined bilateral striatal cluster described in the contrast (Fig. 1). We hypothesized that the change in response time following the trial is an empirical measure of the signal update magnitude. Using Equation 1, we related the observed response magnitude estimates to the behavioral change in response time and the change in probability of stop trials.

### 5.1 Methods

We can validate the model in Equation 1 by testing whether it is predictive of activity during the task. Taking the ROI from Task Contrast Cue period, we measured median post-trial striatal activity between 1 and 5 s following the stop signal delay, and peak activity between 1 and 5 s following the stop signal delay.

In a simple individual difference test, we took subject averages of peak trough activity shown in Figure 4, as well as subject averages of post-pre response time change in response to Stop trials. We then measured the correlation of these subject averages across subjects. In order to reduce effects driven by subjects learning the practical arrangement of the task (such as where to look or attend for various stimuli), we excluded the first 25 trials from analysis.

To test within-subjects effects, we created a series of mixed-effect models predicting median activity from the fixed effects of ΔRT and ΔP(Stop) at two points: the 1-5 s range and the 4-10 s range. These ranges were designed to capture the initial Failed Stop spike visible in Figure 4 as well as the valenced Failed/Correct Stop signal at 4-10 s. Models including ΔP(Stop), ΔRT, or both were included and compared in order to test whether each of these variables significantly predicted variance in the striatal Post-Stop functional ROI. As a confound control, we also included trial number as a fixed effect in each model. This yielded a total of three fixed-effect predictors within each model. In addition, all models which included either ΔP(Stop) and ΔRT as fixed effects also included corresponding random effects varying randomly across subjects.

For the mixed-effects model, the fixed effects are defined as

$$\text{Fixed effects}:y=\beta_{0}+\beta_{1}t+\beta_{2}c+\beta_{3}\Delta \;P\left(Stop\right)+\beta_{4}\Delta \;RT$$

where *t* represents the trial number, *c* is a binary variable representing Failed or Correct Stop, and beta values are the fixed-effects coefficients of the intercepts.

Random effects are

$$\text{Random effects}\;:\;\left(u_{1i}+u_{2i}\Delta \;RT+u_{3i}\Delta \;P\left(Stop\right)+v_{j}\right)$$

where i represents levels of subjects, and j represents levels of waves. We then can model random effects as

$$u_{1i}=\gamma_{1}+\Theta_{1i},u_{2i}=\gamma_{2}+\;\;\Theta_{2i},u_{3i}=\gamma_{3}+\Theta_{3i},\nu_{j}=\delta +\eta_{j}$$

with $\Theta_{ji},\;\eta_{j}$ assumed to be normally distributed with mean 0. Finally, the overall model can be written as:

y=Fixed effects+Random effects+ε

The model was run using R’s lme4 package, and the code for the commands run for each of the models is described in the “Open data supplementary/Linear models” section of the Supplementary Material. R’s anova function was used to measure AIC, BIC, and Log Likelihood for each model, and to perform a chi-squared test of the differences in log likelihoods of the models to determine whether adding parameters for either P(Stop), RT improved model performance.

Overall, there were three sets of models for comparison. The first describes median activity 4-10 s post-stop signal in all stop trials; the second describes activity specifically in Failed Stop trials. The third describes activity in Failed Stop trials 1-5 s following Failed Stop trials. We did not run a model for activity across all stop trials at the 1-5 s mark because the spike at this time period is only observable in the Failed Stop condition (see Fig. 5) None of the activity measured was adjusted with an HRF function.

Data were collected from two waves of the SST task.

### 5.2 Results

In a mixed-effects model predicting post-stop trial activity (median of 4-10 s) (Table 1), both Post-Pre ΔRT (B = -0.05, 95% CI [-0.08, -0.02]) and ΔP(Stop) (B = 1.53, [1.03, 2.04]) were related to median post-trial activity at 4-10 s. Model comparisons showed that a model that included ΔRT (χ^2^ = 43.0, p < 0.001) and ΔP(Stop) (χ^2^ = 11.3, p < 0.001) predicted the outcome significantly more than models that did not include both of those variables. Overall, the full model had a marginal R^2^ of 0.057 and conditional R^2^ of 0.070, indicating 5.7% of fixed effect and 7.0% of all variance explained. The same pattern was evident in a second mixed-effect model (Table 2) describing only activity following Failed Stop trials: both ΔRT (B = -0.05, [-0.09, -0.01]) and ΔP(Stop Trial) (B = 2.16, [1.46, 2.87]) were related to median post-stop-trial activity at 4-10 s. Model comparisons showed that a model of post-Failed Stop activity that included ΔRT (χ^2^ = 34.86, p < 0.001) and ΔP(Stop) (χ^2^ = 5.6, p < 0.05) predicted the outcome significantly more than models that did not include both of those variables. Overall, the full Failed Stop model had a marginal R^2^ of 0.019 and a conditional R^2^ of 0.092, indicating 1.9% of fixed-effect variance and 9.2% of all variance explained.

**Table 1.** Linear model describing median activity at 4-10 s following stop signal in Correct and Failed Stop trials.

Parameter	Dependent variable:		
	Median activity 4-10 s post-stop signal		
	RT only	P(Stop) only	Full model
Fixed effects with 95% confidence intervals			
(Intercept)	0.21 [0.17, 0.25]	-0.33 [-0.51, -0.15]	-0.33 [-0.51, -0.15]
Standardized Trial Number	0.04 [0, 0.07]	-0.2 [-0.29, -0.12]	-0.2 [-0.29, -0.12]
Stop Failure	-0.44 [-0.5, -0.39]	-0.46 [-0.52, -0.41]	-0.45 [-0.51, -0.4]
Standardized Post-Pre ΔRT	-0.05 [-0.08, -0.02]		-0.05 [-0.08, -0.02]
Standardized ΔP(Stop Trial)		1.51 [1.01, 2.02]	1.53 [1.03, 2.04]
Random effects (SE)			
subid.(Intercept)	0	0.18	0.19
subid.Standardized Post-Pre ΔRT	0.03	0.06	0.01
subid.Standardized ΔP(Stop Trial)	0.25	0.74	0.75
wave.(Intercept)	0.01	0.01	0.01
Number of parameters	12	12	13
AIC	15221	15189	15180
BIC	15300	15268	15266
Log Likelihood	-7599	-7583	-7577
Chi-square vs.			
ΔRT Only, Excluding ΔP(Stop)			43.04***
ΔP(Stop) Only, Excluding ΔRT			11.3***

First 25 trials omitted. ***p < 0.001.

**Table 2.** Linear model describing median activity at 4-10 s following the stop signal, only in Failed Stop trials.

Parameter	Dependent variable:		
	Median activity 4-10 s post-stop signal		
	RT only	P(Stop) only	Full model
Fixed effects with 95% confidence intervals			
(Intercept)	-0.25 [-0.36, -0.14]	-1.07 [-1.35, -0.79]	-1.03 [-1.31, -0.75]
Standardized Trial Number	0.04 [-0.01, 0.1]	0.37 [0.25, 0.49]	0.37 [0.25, 0.49]
Standardized Post-Pre ΔRT	-0.06 [-0.09, -0.02]		-0.05 [-0.09, -0.01]
Standardized ΔP(Stop Trial)		2.2 [1.49, 2.9]	2.16 [1.46, 2.87]
Random effects (SE)			
subid.(Intercept)	0.51	0.47	0.47
subid.Standardized Post-Pre ΔRT	0.06	0.07	0.01
subid.Standardized ΔP(Stop Trial)	1.14	0.96	0.99
wave.(Intercept)	0.07	0.08	0.07
Number of parameters	11	11	12
AIC	6533	6504	6500
BIC	6597	6567	6569
Log Likelihood	-3256	-3241	-3238
Chi-square vs.			
ΔRT Only, Excluding ΔP(Stop)			34.86***
ΔP(Stop) Only, Excluding ΔRT			5.6*

First 25 trials omitted. *p < 0.05, ***p < 0.001.

#### 5.2.1 Post-failed stop spike

Examining the post-Failed Stop spike (Table 3), we see that in Failed Stop Trials there is a strong relationship between median spike activity with ΔP(Stop) (B = 0.53, 95% CI [0.2, 0.86]) but not with ΔRT (B = 0.01, [-0.01, 0.03]). Model comparisons showed that relative to models that committed one of these variables compared to the full model, ΔP(Stop) significantly improved model fit (χ^2^ = 7.63, p < 0.01) but including ΔRT did not (χ^2^ = 0.52, p > 0.05). Overall, the full Failed Stop Spike model had a marginal R^2^ of 0.008 and a conditional R^2^ of 0.059, indicating 0.8% of fixed-effect variance and 5.9% of all variance explained.

**Table 3.** Linear model describing median activity at 1-5 s following the stop signal, only in Failed Stop trials.

Parameter	Dependent variable:		
	Median activity 1-5 s post-stop signal		
	RT only	P(Stop) only	Full model
Fixed effects with 95% confidence intervals			
(Intercept)	0.06 [0.02, 0.1]	-0.13 [-0.25, -0.01]	-0.13 [-0.26, -0.01]
Standardized Trial Number	-0.03 [-0.06, -0.01]	0.05 [-0.01, 0.11]	0.05 [-0.01, 0.11]
Standardized Post-Pre ΔRT	0.01 [-0.01, 0.03]		0.01 [-0.01, 0.03]
Standardized ΔP(Stop Trial)		0.53 [0.2, 0.86]	0.53 [0.2, 0.86]
Random effects (SE)			
subid.(Intercept)	0.07	0	0
subid.Standardized Post-Pre ΔRT	0.07	0.07	0.07
subid.Standardized ΔP(Stop Trial)	0.3	0.21	0.21
wave.(Intercept)	0.02	0.02	0.02
Number of parameters	11	11	12
AIC	3055	3048	3050
BIC	3118	3111	3118
Log Likelihood	-1517	-1513	-1513
Chi-square vs.			
ΔRT Only, Excluding ΔP(Stop)			7.63**
ΔP(Stop) Only, Excluding ΔRT			0.52

First 25 trials omitted. **p < 0.01.

### 5.3 Discussion

Confirming the prediction based on the prior section, the analysis in this section shows that the 4-10 s period following the stop signal appears to track learning of both the SSD (operationalized as RT) and P(Stop). The result is more ambiguous for the Failed Stop spike, which does not encode ΔRT, but seems to encode ΔP(Stop).

## 6 Discussion

We examined learning in the SST and found several patterns of activity that are informative about learning processes during the inhibitory control task. These findings suggest that not only can inhibitory control be learned (Verbruggen & Logan, 2008) but also learning of inhibitory control follows a reward prediction error pattern when learning probabilistic cues. To our knowledge, this is the first study to demonstrate co-occurring multiple forms of reinforcement learning being simultaneously processed in the striatum, suggesting that the striatum forms part of a broader RPE system that can learn multiple patterns simultaneously.

Post-error slowing is related to decreases in striatal activity in response to errors. The striatal activation apparent in the contrast between post-stop failed and correct responses can be decomposed into a small post-correct activation and large post-error de-activation, consistent with a model whereby that activity acts as a reward prediction error signal. A mixed-effects model indicates the striatal activity might be a learning signal that encodes reaction time change and the current expected probability of a stop trial occuring. This extends Chevrier and Schacher’s (2010) finding that post-error striatal deactivation follows Failed Stop trials, by linking striatal activity to post-error reaction time change, and provides evidence that the striatum encodes a multivariate reward prediction error signal for learning within the SST.

### 6.1 Relevance of these findings

The Stop Signal Task is a useful tool for measuring learning in individual difference measures response inhibition through behavioral and neural data (White et al., 2014). Two distinct neural signals have been identified following the SST Stop Signal. The first is a peak that follows the Stop Signal and appears to be universal across the striatum, ACC, and FOC. This signal responds differentially to the Stop signal compared to its absence and may function to track the probability of a stop signal, making it a reaction to an unexpected stimulus. It does not cleanly track reward prediction error. The second signal is an RPE signal that can be disentangled from simple valence because it tracks post-pre ΔRT even after controlling for Correct vs. Failed Stop difference. This signal is active in all three parts of the striatum but notably not in the ACC or vmPFC.

### 6.2 Trial-average time courses

We presented fMRI data that circumvented the assumptions inherent to a canonical HRF. The large number of trials and high statistical power of our study afforded these relatively novel analyses. We plotted the BOLD signal directly because we wanted an unfiltered view into how activity changes dynamically over time in response to a stop signal. The corpus of over 200 subjects allows us to measure the group average response with a higher level of precision than would be possible with a smaller dataset. This approach provides a more direct analysis of BOLD signal that is not convolved through an assumed HRF, so the time course of activity following the stop signal must in part represent the shape of the haemodynamic response rather than the neural activity itself. The distinctive patterns identified in response to Correct Stop and Failed Stop trials illustrate that an examination of averages of raw BOLD signals is still (a) useful for setting apart signals in Failed Stop and Correct Stop as well as (b) identifying different functional signals in the data. This approach is a promising way to examine data in cases with large samples.

### 6.3 RPE and the striatum

There is a striking similarity between putamen, accumbens, and caudate in the RPE signal. This suggests all three of these areas are engaged in the signal we detected. If this signal is RPE, this would lend weight to the idea that an RPE signal is carried in all of these areas. There has been active debate in recent years about the role of the sub-components of the striatum in reward and RPE as well as other functions such as in movement. Lerner et al. (2021) have recently emphasized the strong association between RPE and dopamine release, while also identifying a possible role for dopamine in movement, motivation, and goal-directed planning. In an animal Go-NoGo task, Syed et al. (2016) found that nucleus accumbens dopamine was attenuated unless the action involved movement (the interpretive puzzle in the present study is the broad set of regions, including the nucleus accumbens, that spike after a failure but not a success). Situated within this research context, the present work (Fig. 5) adds support to the proposition that all regions of the striatum appear to be engaged in RPE-related activity, but that these regions are *also* implicated in activity not clearly related to task-related RPE.

### 6.4 Limitations

A *reward* signal indicates an absolute presence of a reward, while a reward prediction error indicates a positive or negative discrepancy between expected and observed reward. It is sometimes difficult to fully disentangle reward prediction error from simple reward signals (Niv, 2009). This is true in the case of the SST, where RPE correlates strongly with simple valenced positive and negative rewards, but is not otherwise directly quantified. This is why we have used proxies to quantify RPE, including ΔP(Stop) and SSD. Whether the observed striatal signal is RPE or simply reward, a distinctive signal tracking both P(Stop) Change and SSD Change is a novel observation in the literature around the SST and possibly in learning tasks in general. We observed the presence of change—distinct from the absolute values—of P(Stop) and SSD in post-stop striatal signal, and the specificity of this finding is suggestive of an RPE interpretation over a simple reward interpretation. Finally, Lerner et al. (2021) suggest that RPE can be observed in the striatal activity during learning tasks.

### 6.5 First post-stop signal

The Failed Stop spike could be characterized as salience, surprise, or error-related negativity. We identified an activity peak immediately following a failed stop signal, but not a successful stop signal. The activity peak was visible across all regions examined: ACC, vmPFC, and the striatum. The activity did not appear to be correlated with change in reaction times, so there is no evidence it is an RPE for learning. There was a weak correlation with predicted P(Stop) change, though considering the very high correlation of P(Stop) and trial, a weak correlation between P(Stop) change and activity seems possibly spurious.

## 7 Conclusion

This article presents evidence that the striatum tracks an RPE signal during the SST, and that the signal comprised learning about two distinct task parameters: P(Stop) and the Stop Signal Delay. It also presents evidence of a *separate* signal, observable only in response to failed stop trials. Finally, this article demonstrates the usefulness of a Trial average BOLD time course design in a sample of over 200 subjects.

## Supplementary Material

Supplementary Material

## Data Availability

The code for analyses in this paper is available at https://github.com/UOSAN/DEV_scripts, and guidelines for using the files within that repository to reproduce the analyses are available in the Supplementary Material. Level 2 SPM analyses and source files for contrasts presented in Section 3 are available for Stop trials here available here, for intervals following Stop trials at available here, and for Go trials following Stop trials at available here. Data presented in Sections 4 and 5 can be fully reproduced from codes available at this link and ROI and behavioral data can be downloaded at https://files.osf.io/v1/resources/zuqyg/providers/osfstorage/649dfba567aff80ee0edfae5/?zip= More detail is provided in the Supplementary Material. To obtain raw fMRI data for generating ROIs, please contact the corresponding author.
