# Supplementary material for: Striatal response to negative feedback in a stop signal task operates as a multi-value learning signal
Source: Imaging Neurosci (Camb). Author manuscript; Available in PMC 2023 Dec 4. (PMC10695358; doi:10.1162/imag_a_00024)
Supplement: 2 [file NIHMS1946108-supplement-2.pdf]

Supplemental material

Method supplementary

Neuroimaging sequence parameters

We acquired a high-resolution anatomical T1-weighted MP-RAGE scan (TR/TE = 2500.00/3.43ms, 256 × 256 matrix, 1mm thick, 176 sagittal slices, FOV = 208 × 208mm), functional images with a T2*- weighted echo-planar sequence (72 axial slices, TR/TE = 2000.00/27.00ms, 90-degree flip angle, 100 × 100 matrix, 2mm thick, FOV = 208 × 208mm, multiband acceleration factor = 3), and opposite phase encoded echo-planar images to correct for magnetic field inhomogeneities (72 axial slices, TR/TE = 6390.00/47.80ms, 90-degree flip angle, 104 ×104 matrix, 2mm thick, FOV = 208 × 208mm).

Initial pre-processing

DICOMS were converted into BIDS format using the bidsQC package (https://github.com/kdestasio/bidsQC) which wraps the dcm2Bids project.

fMRIPrep preprocessing

Results included in this manuscript come from preprocessing performed using fMRIPrep 22.1.1 (Esteban, Markiewicz, et al. (2018); Esteban, Blair, et al. (2018); RRID:SCR_016216), which is based on Nipype 1.8.5 (K. Gorgolewski et al. (2011); K. J. Gorgolewski et al. (2018); RRID:SCR_002502).

Preprocessing of B0 inhomogeneity mappings

A B0-nonuniformity map (or fieldmap) was estimated based on all echo-planar imaging (EPI) references available (usually 2) with topup (Andersson, Skare, and Ashburner (2003); FSL 6.0.5.1:57b01774).

Anatomical data preprocessing

T1-weighted (T1w) images were corrected for intensity non-uniformity (INU) with N4BiasFieldCorrection (Tustison et al. 2010), distributed with ANTs 2.3.3 (Avants et al. 2008, RRID:SCR_004757). The T1w-reference was then skull-stripped with a Nipype implementation of the antsBrainExtraction.sh workflow (from ANTs), using OASIS30ANTs as target template. Brain tissue segmentation of cerebrospinal fluid (CSF), white-matter (WM) and gray-matter (GM) was performed on the brain-extracted T1w using fast (FSL 6.0.5.1:57b01774,

RRID:SCR_002823, Zhang, Brady, and Smith 2001). A T1w-reference map was computed after registration of all available (typically 2) T1w images (after INU-correction) using mri_robust_template (FreeSurfer 7.2.0, Reuter, Rosas, and Fischl 2010). Brain surfaces were reconstructed using recon-all (FreeSurfer 7.2.0, RRID:SCR_001847, Dale, Fischl, and Sereno 1999), and the brain mask estimated previously was refined with a custom variation of the method to reconcile ANTs-derived and FreeSurfer-derived segmentations of the cortical gray-matter of Mindboggle (RRID:SCR_002438, Klein et al. 2017). Volume-based spatial normalization to one standard space (MNI152NLin2009cAsym) was performed through nonlinear registration with antsRegistration (ANTs 2.3.3), using brain-extracted versions of both T1w reference and the T1w template. The following template was selected for spatial normalization: ICBM 152 Nonlinear Asymmetrical template version 2009c [Fonov et al. (2009), RRID:SCR_008796; TemplateFlow ID: MNI152NLin2009cAsym].

Functional data preprocessing

For each run, the following preprocessing was performed. First, a reference volume and its skull-stripped version were generated using a custom methodology of fMRIPrep. Head-motion parameters with respect to the BOLD reference (transformation matrices, and six corresponding rotation and translation parameters) are estimated before any spatiotemporal filtering using mcflirt (FSL 6.0.5.1:57b01774, Jenkinson et al. 2002). The estimated fieldmap was then aligned with rigid-registration to the target EPI (echo-planar imaging) reference run. The field coefficients were mapped on to the reference EPI using the transform. The BOLD reference was then co-registered to the T1w reference using bbregister (FreeSurfer) which implements boundary-based registration (Greve and Fischl 2009). Co-registration was configured with six degrees of freedom. Several confounding time-series were calculated based on the preprocessed BOLD: framewise displacement (FD), DVARS and three region-wise global signals. FD was computed using two formulations following Power (absolute sum of relative motions, Power et al. (2014)) and Jenkinson (relative root mean square displacement between affines, Jenkinson et al. (2002)). FD and DVARS are calculated for each functional run, both using their implementations in Nipype (following the definitions by Power et al. 2014). The three global signals are extracted within the CSF, the WM, and the whole-brain masks. Additionally, a set of physiological regressors were extracted to allow for component-based noise correction (CompCor, Behzadi et al. 2007). Principal components are estimated after high-pass filtering the preprocessed BOLD time-series (using a discrete cosine filter with 128s cut-off) for the two CompCor variants: temporal (tCompCor) and anatomical (aCompCor). tCompCor components

are then calculated from the top 2% variable voxels within the brain mask. For aCompCor, three probabilistic masks (CSF, WM and combined CSF+WM) are generated in anatomical space. The implementation differs from that of Behzadi et al. in that instead of eroding the masks by 2 pixels on BOLD space, a mask of pixels that likely contain a volume fraction of GM is subtracted from the aCompCor masks. This mask is obtained by dilating a GM mask extracted from the FreeSurfer's aseg segmentation, and it ensures components are not extracted from voxels containing a minimal fraction of GM. Finally, these masks are resampled into BOLD space and binarized by thresholding at 0.99 (as in the original implementation). Components are also calculated separately within the WM and CSF masks. For each CompCor decomposition, the k components with the largest singular values are retained, such that the retained components' time series are sufficient to explain 50 percent of variance across the nuisance mask (CSF, WM, combined, or temporal). The remaining components are dropped from consideration. The head-motion estimates calculated in the correction step were also placed within the corresponding confounds file. The confound time series derived from head motion estimates and global signals were expanded with the inclusion of temporal derivatives and quadratic terms for each (Satterthwaite et al. 2013). Frames that exceeded a threshold of 0.5 mm FD or 1.5 standardized DVARS were annotated as motion outliers. Additional nuisance timeseries are calculated by means of principal components analysis of the signal found within a thin band (crown) of voxels around the edge of the brain, as proposed by (Patriat, Reynolds, and Birn 2017). The BOLD time-series were resampled into standard space, generating a preprocessed BOLD run in MNI152NLin2009cAsym space. First, a reference volume and its skull-stripped version were generated using a custom methodology of fMRIPrep. The BOLD time-series were resampled onto the following surfaces (FreeSurfer reconstruction nomenclature): fsaverage, fsnative. All resamplings can be performed with a single interpolation step by composing all the pertinent transformations (i.e. head-motion transform matrices, susceptibility distortion correction when available, and co-registrations to anatomical and output spaces). Gridded (volumetric) resamplings were performed using antsApplyTransforms (ANTs), configured with Lanczos interpolation to minimize the smoothing effects of other kernels (Lanczos 1964). Non-gridded (surface) resamplings were performed using mri_vol2surf (FreeSurfer).

Many internal operations of *fMRIPrep* use *Nilearn* 0.9.1 (Abraham et al. 2014, RRID:SCR_001362), mostly within the functional processing workflow. For more details of the pipeline, see the section corresponding to workflows in *fMRIPrep*'s documentation.

Further preprocessing

fMRIPrep output was manually examined for scan errors. Data from 10 subjects excluded due to moderate or severe ghosting or other distortions.

Prior to first-level modeling, we generated motion regressors using an automated motion assessment tool (Cosme et al., 2018). This tool applies a predictive model that utilizes the confound files generated by fMRIPrep and classifies whether or not fMRI volumes contain motion artifacts. The classifier is applied to each participant's task run and returns a binary classification indicating the presence or absence of motion artifacts for each volume. In addition, this tool transforms the realignment parameters into Euclidean distance for translation and rotation separately, and calculates the displacement derivative of each. This procedure yields a total of 5 motion regressors for first-level modeling. Task runs that contain >10% of volumes classified as containing a motion artifact will be excluded from further analyses.

Finally, 6mm FWHM smoothing was applied using SPM12's spatial smoothing tool.

First-level modeling

Event-related condition effects were estimated in first-level analyses using a fixed-effects general linear model and a canonical hemodynamic response function. Regressors modeled each experimental condition as described below. Trials were measured from the onset of the arrow to the disappearance of the arrow, irrespective of the participant's reaction time. Additional regressors of no interest were added for the instruction and rating periods. Five motion regressors were modeled as covariates of no interest. Realignment parameters were transformed into Euclidean distance for translation and rotation separately. Data were high-pass filtered at 128 seconds and temporal autocorrelation was modeled using FAST (Corbin et al., 2018).

There were three first-level models. Each model classified each trial into a set of trials and contained one regressor for each of them, in addition to several motion regressor and a constant regressors. The simplest model was designed to examine activity between the two trial types crossed with the two possible participant responses, i.e., Correct Stop, Failed Stop, Correct Go, Failed Go. It also included a Cue regressor to capture activity in between trials, five motion regressors, and a constant regressor. The design is depicted below. The first-level analysis included bidirectional contrasts between Failed and Correct Stop regressors.

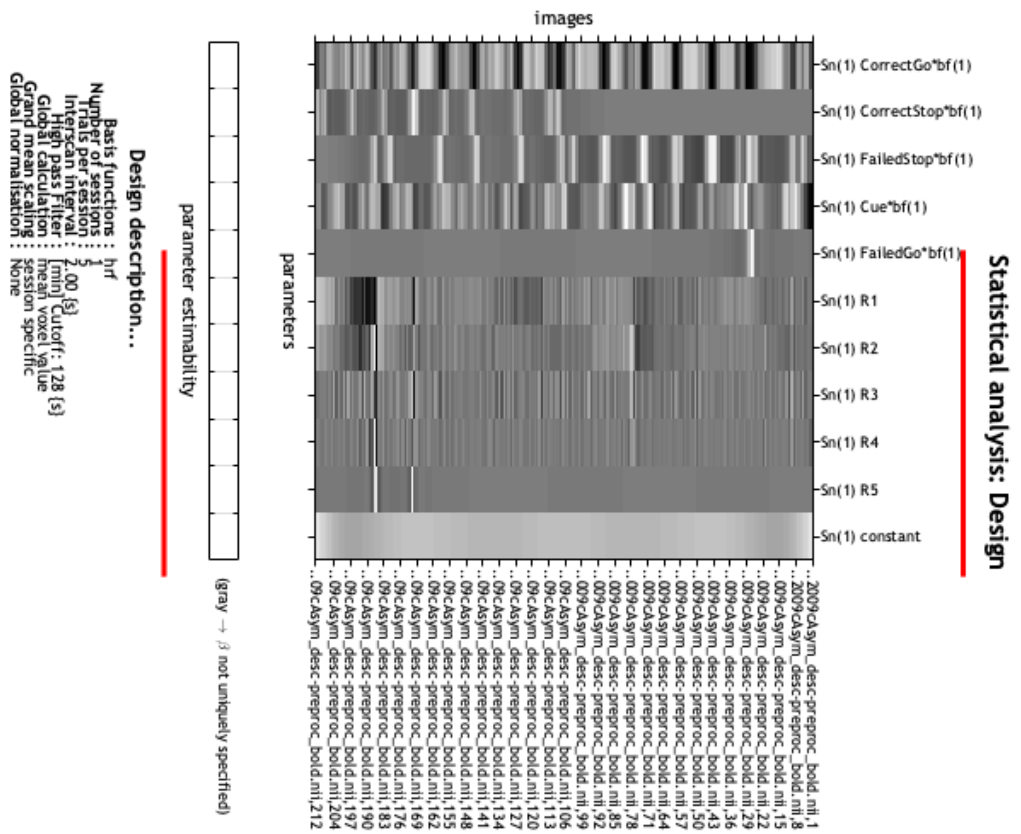

The second model was designed to facilitate contrasts between trials classified based on the trial that preceded them. It extends the first model, but differs in several respects. First, Correct Go trials were separated into separate regressors depending on whether they followed a Correct Stop, a Failed Stop, or another trial type. Second, Correct Stop and Failed Stop trails were each separated into separate regressors depending on whether they preceded a Correct Go, or some other trial. One particular subject is depicted below. The first-level analysis included bidirectional contrasts between regressors for Go trials following either Failed or Correct Stop trials.

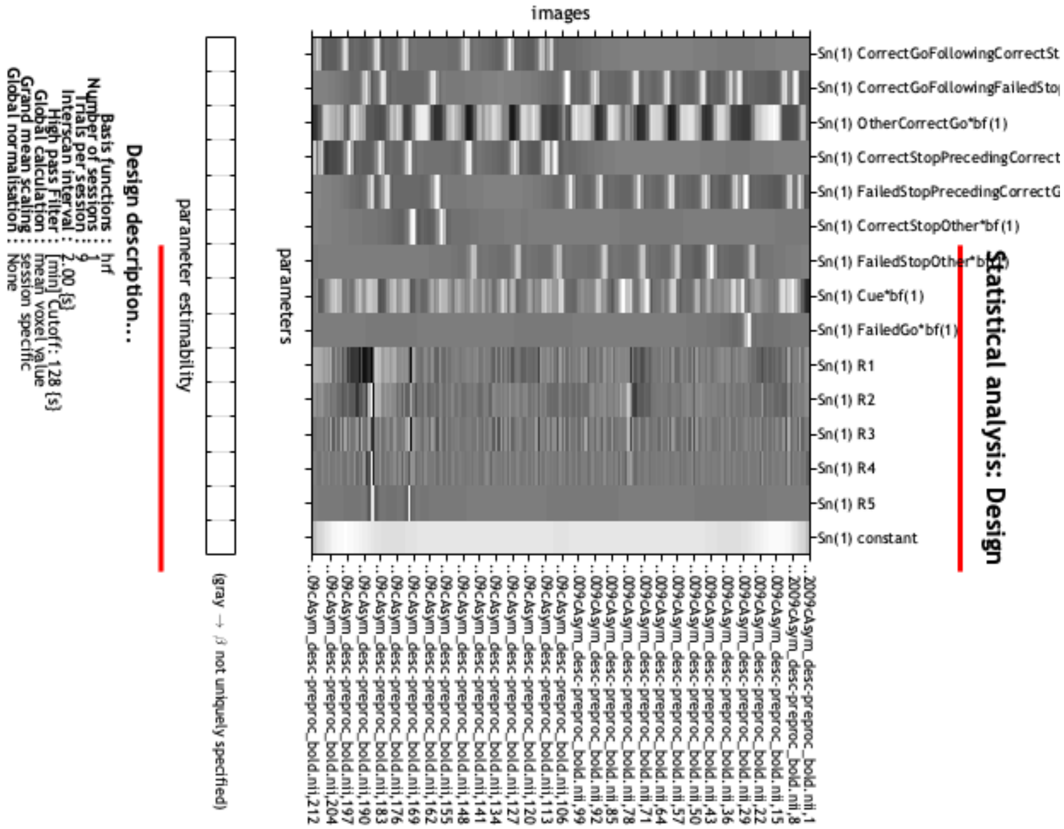

The third model was designed to facilitate contrasts between cues classified based on the trial that preceded them. This model extended the second model, but like Correct Go trials, cues were also separated into distinct regressors depending on whether they followed a Correct Stop, Failed Stop, or some other trial type. The design for one particular subject is depicted below. The first-level analysis included bidirectional contrasts between regressors for Cues following either Failed or Correct Stop trials.

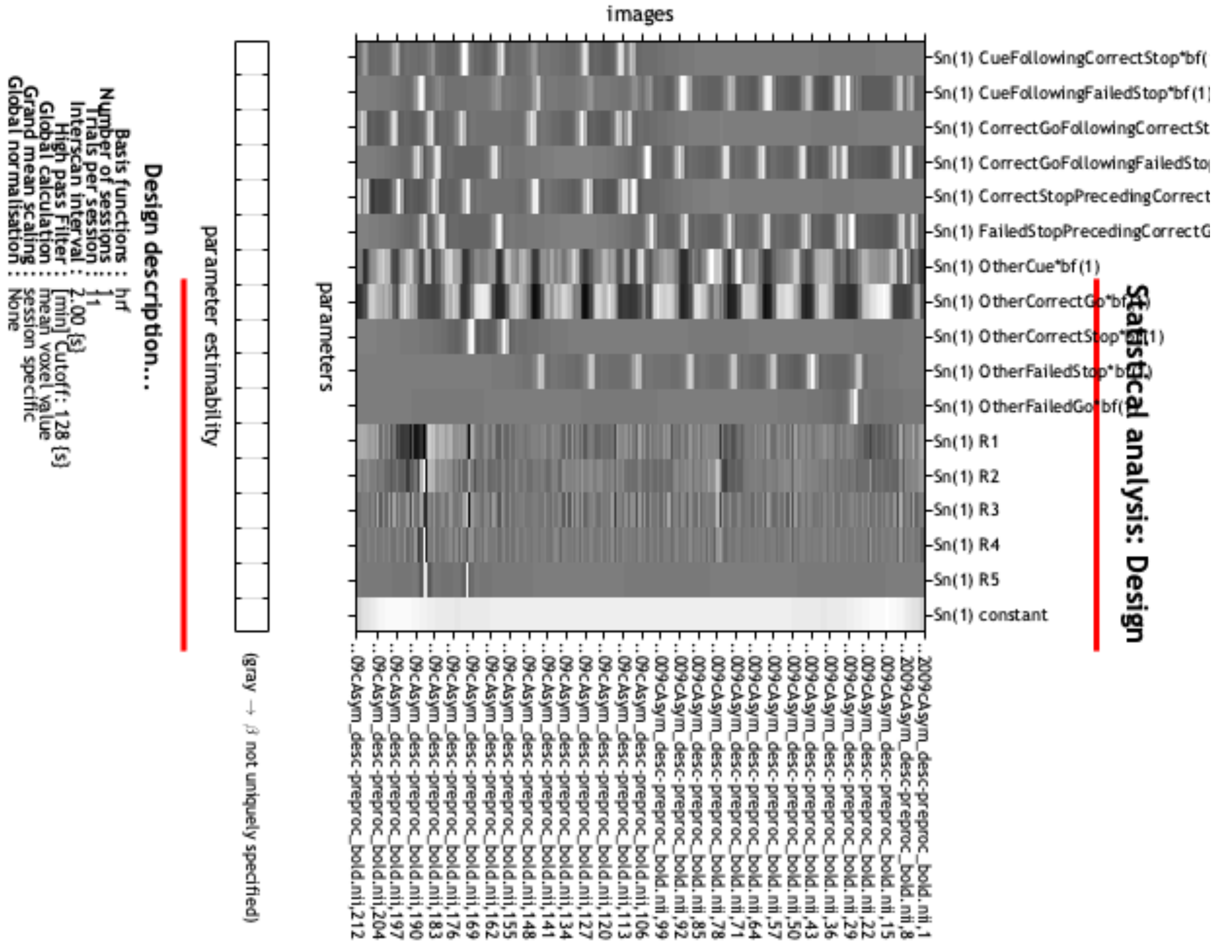

Removing first 25 trials

We considered whether to remove the first 25 trials from each run. During these trials, it's possible participants are learning things other than P(Stop) and Post-Pre ΔRT, such as just learning how the task works in the first place. Subsequently, P(Stop) should stabilize but participants should continue to learn how post-pre RT change over time. Consequently, we removed the first 25 trials from analyses in this section.

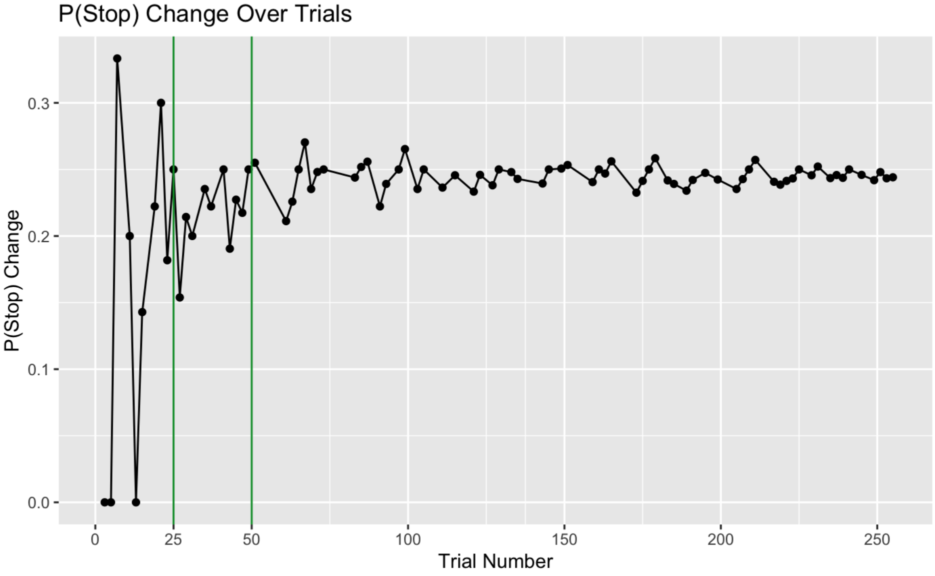

Variance on additional trials and subject exclusions

In follow-up testing, we tested results with early trials (up to 25) excluded. The results varied substantially depending on whether early trials were excluded, and the significance of post-error slowing and P(Stop) became marginal in some cases. It may be that most of the learning observed occurs in early trials.

We also examined the proportion of Correct Stop trials in each run. Those are displayed in the figure below. Runs where subjects scored less than 20% correct or more than 80% were excluded, as these values were implausible, considering that the task was calibrated to achieve roughly 50% performance. Overall, 25 runs of 411 were removed, 23 because their proportion of stop trials correct was under 20%, and 2 because it was over 80%. This included 5 subjects whose runs were all removed, leaving 212 subjects for the time course and multi-level model analyses.

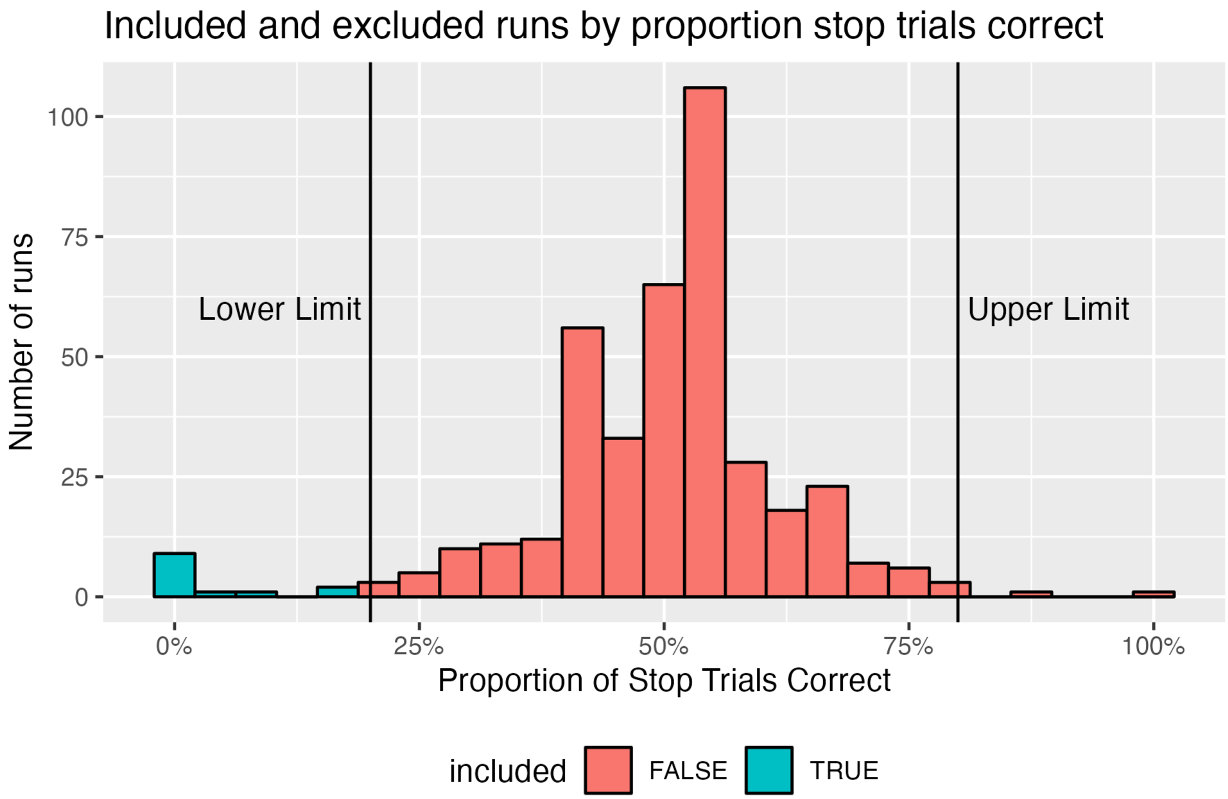

Variance on including additional trials

Open data supplementary

The code used to perform all analyses and generate tables and figures across Analyses B-C is available in https://github.com/UOSAN/DEV_scripts/blob/master/fMRI/fx/models/SST/direct_regression/sst_paper_generation_w12.Rmd. The code to generate the Task Contrast is described below. Contact the authors to discuss access to the raw data to replicate these findings.

Level 1

This section describes the data flow in the raw files.

First we generate the following 1st-level SPM scripts:

- DEV_scripts/fMRI/fx/models/SST/batch_spm_job_fx.sh, which runs:
    - DEV_scripts/fMRI/fx/models/SST/fx_conditions_w1_w_contrasts.m

- DEV_scripts/fMRI/fx/models/SST/batch_spm_job_fx_posterror_conditions.sh, which runs one of two different SPM contrasts depending on data available:
  - DEV_scripts/fMRI/fx/models/SST/fx_posterror_conditions_w1_w_contrasts.m
  - DEV_scripts/fMRI/fx/models/SST/fMRI/fx/models/$TASK/fx_posterror_conditions_w1.m
- And this SPM contrast fx_conditions_w1_w_cues_post_pre.m which is run by batch_spm_job_fx.sh

We also need to generate timing files:

- full_duration/posterror_cues_no_rt
- full_duration/conditions (generated by multiconds_full_duration.py)
- full_duration/posterror_conditions

Then, these create these level 1 contrasts:

- DEV/nonbids_data/fMRI/fx/models/SST/wave1/posterror_cues_no_rt/
- DEV/nonbids_data/fMRI/fx/models/SST/wave1/conditions/
- DEV/nonbids_data/fMRI/fx/models/SST/wave1/posterror_conditions/

Level 2

Before we do level 2, might need to copy those items off the server onto a local machine where it's easier to do using rsync. An example of a copy command might look like:

- rsync -av user@talapas-ln2.uoregon.edu:/gpfs/projects/sanlab/shared/DEV/nonbids_data/fMRI/fx/models/SST/wave1/posterror_cues_no_rt/ "/DEV/nonbids_data/fMRI/fx/models/SST/wave1/posterror_cues_no_rt/" --exclude='archive' --include='sub-DEV*/spmT*' --include='sub-DEV*/con_*' --include='sub-DEV*/beta_000[0-9].nii' --include='sub-DEV*/beta_001[0-2].nii' --exclude='sub-DEV*/*.nii'
- This would need to be repeated for each of the three contrast folders

Then we generate level 2 files.

- *level2/posterror_cues_no_rt* generated by DEV_scripts/fMRI/fx/models/SST/level2/generate_one_sample_t_test_posterror_cues_no_rt.py
- level2/conditions generated by */DEV_scripts/fMRI/fx/models/SST/level2/generate_one_sample_t_test_cg_cs.py*
- level2/posterror generated by *DEV_scripts/fMRI/fx/models/SST/level2/generate_one_sample_t_test_posterror.py*

Contrasts

Contrasts in Figure 1 were derived from level 2 contrasts:

- *DEV_scripts/fMRI/fx/models/SST/level2/posterror_cues_no_rt_20230110/CueFollowing(CS>FS)*
- DEV_scripts/fMRI/fx/models/SST/level2/conditions_20221207/CS>FS
- *DEV_scripts/fMRI/fx/models/SST/level2/posterror_20221207/CorrectGoFollowing(CS>FS)*

ROI

We extract the two striatal regions areas of activity from the nii file in /Users/benjaminsmith/Google Drive/oregon/code/DEV_scripts/fMRI/fx/models/SST/level2/posterror_cues_no_rt_20230512/CueFollowing(CS>FS)/spmT_0001.nii using matlab SPM.

Open the SPM.mat file in the results window. After estimating the Effect with the SPM file, I select the first striatal ROI. Then in the Results window, in the Display tab, Save -> current cluster. I then repeat this for the second striatal ROI.
The ROI was generated using the script DEV_scripts/fMRI/fx/models/SST/direct_regression/create_striatal_roi.py

The mask created is then manually copied to the server path nonbids_data/fMRI/ml/masks/sst_functional/

Then DEV_scripts/fMRI/fx/models/SST/direct_regression/get_all_series_main.py creates the ROI activity measurements, including the functional ROI. (this can be launched via bash using get_all_series_main.sh, in the same folder; use `sbatch get_all_series.sh` to schedule as a job)

These are saved in /gpfs/projects/sanlab/bsmith16/data/roi_data_raw.pkl and SST_roi_by_time_point.csv in the same folder.

All graphs and tables can be generated from data

Following that, the graphs and tables are created using DEV_scripts/fMRI/fx/models/SST/direct_regression/sst_paper_generation_w12.Rmd.

Linear models

Table 1 linear model was

```
lme4::lmer(
    med_post_trial_z ~ trial_n_s + condition + P_stop_trial_change_z +
post_pre_rt_change_z + (1 + post_pre_rt_change_z + P_stop_trial_change_z | subid) + (1 |
wave),
   trial_neural_behav_roi %>% filter(trial_n>50 & condition %in%
c("CorrectStop","FailedStop"))
   )
```

Table 2 linear model was

```
lme4::lmer(
    med_post_trial_z ~ trial_n_z + post_pre_rt_change_z + P_stop_trial_change_z +
(1 + post_pre_rt_change_z + P_stop_trial_change_z | subid) + (1 | wave)
,
  trial_neural_behav_roi %>% filter(trial_n>50 & condition %in% c("FailedStop"))
  )
```

Table 3 linear model was

```
lme4::lmer(
    med_post_trial_1_5 ~ trial_n_z + post_pre_rt_change_z + P_stop_trial_change_z +(1+
post_pre_rt_change_z + P_stop_trial_change_z  | subid) + (1 | wave),
  trial_neural_behav_roi %>% filter(trial_n>50 & condition %in% c("FailedStop"))
  )
```

Age and sex

Correct Stop > Failed Stop [Without Control]

During Trial

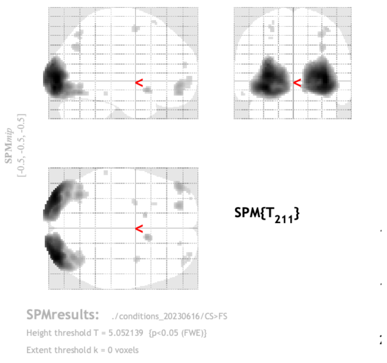

ITI Following Trial

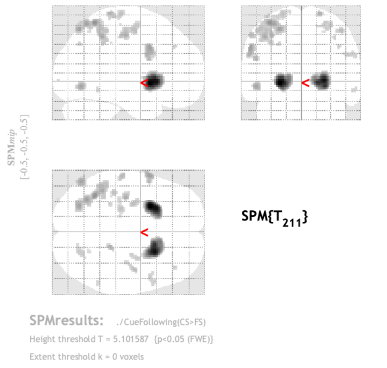

Next Trial

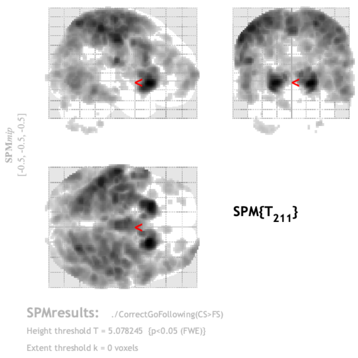

Correct Stop > Failed Stop [With Sex & Age Control]

During Trial

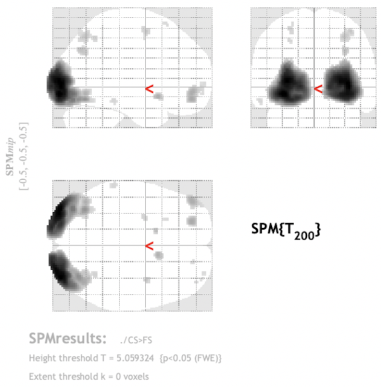

ITI Following Trial

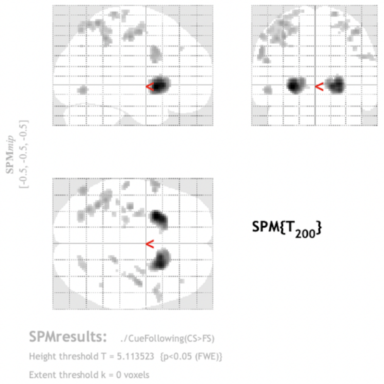

Next Trial

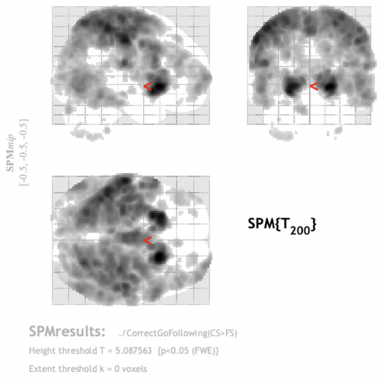

Age [higher]

During Trial | **ITI Following Trial** | **Next Trial**

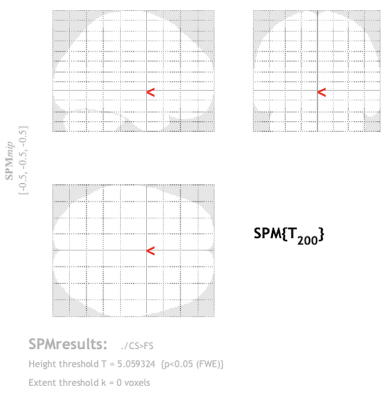

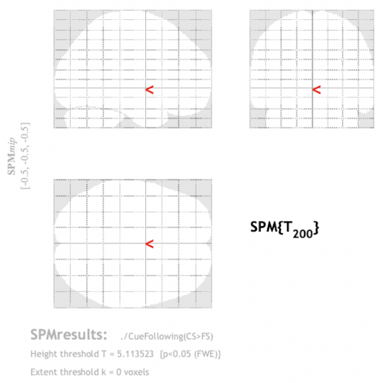

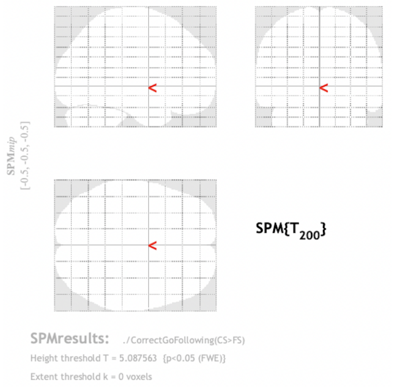

Sex [Male]

Female subjects made up the majority of the dataset, so if there are any Sex differences, base results are more likely to reflect Female patterns. Consequently we treated Female as baseline and tested the effect of Sex=Male

During Trial	ITI Following Trial	Next Trial
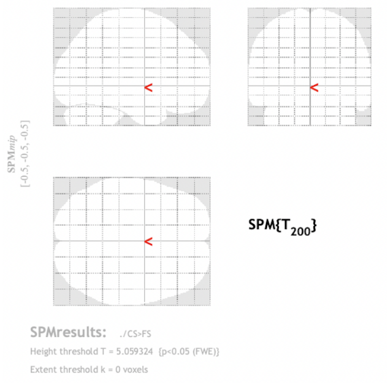	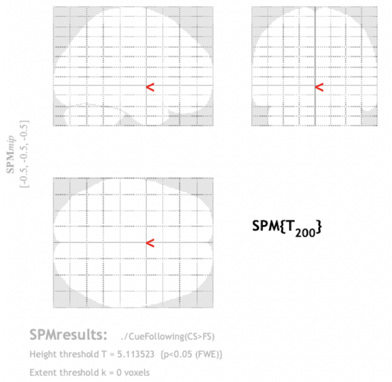	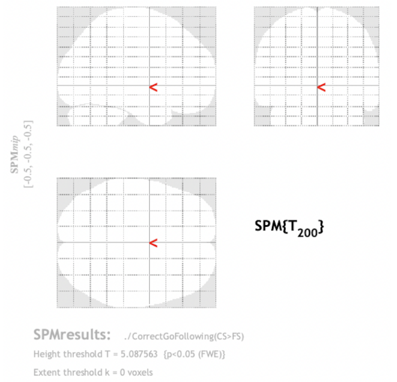
